# Os odontoideum: à propos d'un cas et revue de la littérature

**Published:** 2012-10-30

**Authors:** Mehdi Laghmari, Wafae El Hymer, Khalid Aniba, Mohamed Lmejjati, Houssine Ghannane, Said Ait Benali

**Affiliations:** 1 Département de Neurochirurgie, Université Cadi Ayyad, UHC Mohammed VI, Marrakech, Maroc

**Keywords:** Charnière cervico-occipitale, torticolis, os odontoideum, Cervico-occipital hinge, torticollis, bone odontoideum

## Abstract

L'os odontoideum ou apophyse odontoïde mobile est une malformation rare de la charnière cervico-occipitale (MCCO) qui met en jeu le pronoctic vital et fonctionnel par le risque de compression de la junction bulbo-médullaire. Nous rapportons le cas d'un patient âgé de 22 ans, victime d'un traumatisme cervical à l’âge de 2 ans, chez qui cette affection a été révélée par des torticolis récidivants, puis par l'installation d'une tétraparésie. Bien qu'une symptomatologie déficitaire soit fréquemment révélatrice, la notion de cervicalgie récidivante (même isolée) doit attirer l'attention. Le bilan fait appel aux clichés radiographiques dynamiques, à la TDM et l'IRM. L’étiopathogénie reste méconnue congénitale ou traumatique. Le traitement est chirurgical chez les patients symptomatiques et repose sur l'arthrodèse et l'ostéosynthèse postérieure, tandis que chez les sujets asymptomatiques une simple surveillance est préconisée. Un diagnostic et un traitement précoces permettent d'obtenir une stabilisation et une amélioration clinique dans la majorité des cas.

## Introduction

L'apophyse odontoïde mobile ou os odontoideum fait partie des nombreuses anomalies de la charnière cervico-occipitale. Nous rapportons le cas d'un patient chez qui ce diagnostic a été retenu. A travers cette observation illustrative, nous rappelons les aspects cliniques, étiologiques, radiologiques et pronostiques de cette rare affection et insistons sur l'importance d'un diagnostic précoce, en vue d'une prise en charge adéquate.

## Patient et observation

Un jeune homme de 22 ans ayant comme antécédents une tuberculose abdominale traitée et une chute à l’âge de 2ans occasionnant chez lui un traumatisme du rachis cervical, a présenté depuis deux ans des cervicalgies récidivantes avec torticolis persistant et installation depuis un an d'une lourdeur des quatre membres. L'examen clinique a objectivé une tétraparésie spastique avec amyotrophie intéressant les groupes musculaires des quatre membres sans troubles sensitifs associés.

**Figure 1:**
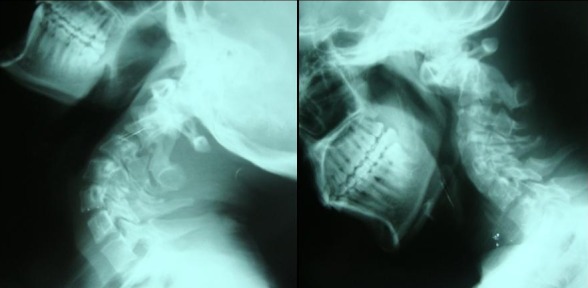
Radiographie dynamique cervicale de profil en flexion extension montrant l'instabilité C1-C2

**Figure 2:**
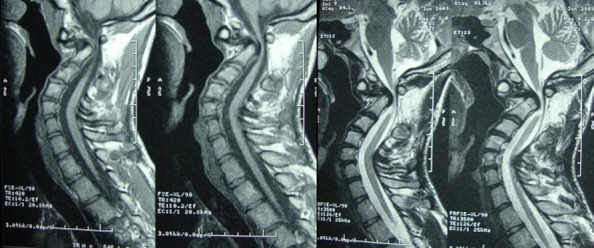
IRM cervicale en coupes sagittales en séquences pondérées T1 et T2 montrant la compression de la jonction bulbo-médullaire par la bascule postérieure du corps de C2 désolidarisé de l'odontoïde

La radiographie standard cervicale a montré une hyper lordose du rachis cervical avec luxation odontoidoaxoidienne (Figure 1). Une IRM cervicale a confirmé la luxation entre l'atlas et l'odontoïde responsable d'une compression majeure de la jonction bulbo médullaire (Figure 2).

**Figure 3:**
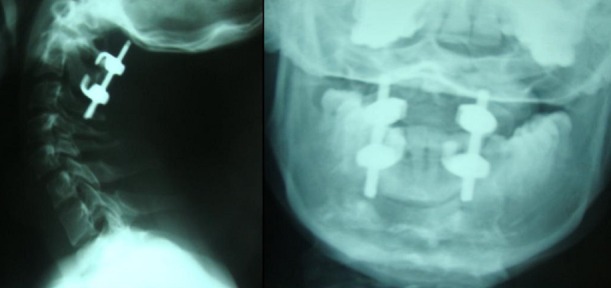
Radiographie centrée sur C2 de profil et de face montrant l'ostéosynthèse par crochets lamaires et tiges en compression réduisant et stabilisant la charnière cervico-occipitale

Le patient a été opéré en position ventrale sous traction transcranienne. Le geste chirurgical a consisté en une dissection des arcs postérieurs de C1 et C2 avec ostéosynthèse postérieure par des crochets sus laminaires en C1 et sous laminaires en C2 montés en compression sur des tiges (Vertex, Medtronic^®^). L'arthrodèse a été réalisée à l'aide de copeaux osseux provenant des épines de C1 et C2 interposés entre les lames de C1 et C2 préalablement ravivées. Les suites post opératoires étaient simples et le contrôle radiologique a montré une parfaite réduction de la luxation (Figure 3).

## Discussion

En raison de la complexité du développement anatomique de la charnière cervico-occipitale et de la transition entre le tronc cérébral et la moelle épinière, la jonction cervico-occipitale peut être le siège d'une multitude d'anomalies osseuses acquises ou congénitales. L'os odontoideum en est une des rares étiologies.

Décrite pour la première fois par Giacomini en1886, ce terme désigne un petit os indépendant siégeant en position craniale par rapport à l'axis, à la place de l'apophyse odontoïde [1]. La pathogénie de cette affection reste un sujet de débat puisque, pour certains auteurs, elle serait d'origine embryonnaire [2], tandis que pour d'autres, elle serait d'origine vasculaire ou encore traumatique [2–5]. C'est le cas de notre patient qui était victime d'un traumatisme à l’âge de 2 ans. Dans ce cas, le traumatisme est assez ancien pour permettre des remaniements de l'apophyse odontoïde qui la transforment en ossicule corticalisé.

Certaines malformations peuvent être associées à l'apophyse odontoïde mobile telle que l'occipitalisation de C1, l'impression basilaire, la maladie de Klippel Feil …Elles sont souvent retrouvées en cas de trisomie 21 [6].

L’âge de découverte est variable. L'affection peut rester longtemps asymptomatique, voire ne jamais se manifester (découverte fortuite), ou causer une mort subite sans que le diagnostic ne soit reconnu.

Sur le plan clinique, l'apophyse odontoïde mobile peut être révélée par des cervicalgies hautes, une faiblesse ou une raideur du cou, un torticolis, des vertiges, une impotence fonctionnelle motrice ou sensitive. Comme chez notre patient, la notion de cervicalgies récidivantes doit attirer l'attention, même en l'absence de signes déficitaires. L'examen neurologique peut révéler un tableau de myélopathie, de radiculopathie, ou encore une atteinte des paires crâniennes [7]. Le bilan doit comporter des radiographies standards du rachis cervical et de la charnière cervico-occipitale. L'os odontoideum apparaît sous forme d'un osselet arrondi ou ovoïde, séparé de la base de l'odontoïde. Contrairement aux fractures récentes de l'apophyse odontoïde où la corticale paraît rompue, l'osselet est corticalisé. Idéalement, des clichés dynamiques (actifs en flexion et extension) sous contrôle médical seront effectués.

La TDM permet une excellente étude des structures osseuses de la charnière cervico-occipitale, tandis que l'IRM met en évidence les répercussions sur la jonction bulbo médullaire [8]. A noter que la sévérité des signes cliniques n'est pas toujours corrélée au degré d'instabilité atloidoaxoidienne [4, 9].

Chez les sujets symptomatiques, le traitement repose sur la chirurgie dont le but est la réduction de la lordose et la stabilisation. En l'absence de réduction on fait un abord postérieur (fusion atloidoaxoidienne ou occipitocervicale), ou un abord transoral [2, 3, 9]. La voie d'abord permettant de lever la compression bulbomédullaire et le rétablissement de l'alignement vertébral normal est la voie postérieure. Une rééducation fonctionnelle est bien entendu indispensable.

Chez les sujets asymptomatiques, l'indication chirurgicale prophylactique est discutée en fonction des données des clichés dynamiques. Toutefois, la majorité des auteurs préconisent une simple surveillance clinique et/ou radiologique régulière. Dans ces cas, les sujets doivent être avertis des risques de complication de cette affection afin d’éviter toute situation à risque.

## Conclusion

L'os odontoideum est une MCCO rare, potentiellement grave. Le diagnostic est basé sur la clinique et des examens radiologiques. Le traitement chirurgical est efficace, basé sur un abord postérieur si la réduction a été réalisée ou une décompression par voie antérieure ou postérieure. Le pronostic dépend de la précocité de la prise en charge.
